# Nerve regeneration in transplanted organs and tracer imaging studies: A review

**DOI:** 10.3389/fbioe.2022.966138

**Published:** 2022-08-16

**Authors:** Yan Huang, Zhigang He, Anne Manyande, Maohui Feng, Hongbing Xiang

**Affiliations:** ^1^ Tongji Hospital of Tongji Medical College, Huazhong University of Science and Technology, Wuhan, Hubei, China; ^2^ Department of Interventional Therapy, the First Affiliated Hospital of Dalian Medical University, Dalian, Liaoning, China; ^3^ School of Human and Social Sciences, University of West London, London, United Kingdom; ^4^ Department of Gastrointestinal Surgery, Wuhan Peritoneal Cancer Clinical Medical Research Center, Zhongnan Hospital of Wuhan University, Hubei Key Laboratory of Tumor Biological Behaviors and Hubei Cancer Clinical Study Center, Wuhan, Hubei, China

**Keywords:** organ transplantation, nerve tracers, sympathetic nerve, parasympathetic nerve, visceral sensory plexus

## Abstract

The technique of organ transplantation is well established and after transplantation the patient might be faced with the problem of nerve regeneration of the transplanted organ. Transplanted organs are innervated by the sympathetic, parasympathetic, and visceral sensory plexuses, but there is a lack of clarity regarding the neural influences on the heart, liver and kidneys and the mechanisms of their innervation. Although there has been considerable recent work exploring the potential mechanisms of nerve regeneration in organ transplantation, there remains much that is unknown about the heterogeneity and individual variability in the reinnervation of organ transplantation. The widespread availability of radioactive nerve tracers has also made a significant contribution to organ transplantation and has helped to investigate nerve recovery after transplantation, as well as providing a direction for future organ transplantation research. In this review we focused on neural tracer imaging techniques in humans and provide some conceptual insights into theories that can effectively support our choice of radionuclide tracers. This also facilitates the development of nuclear medicine techniques and promotes the development of modern medical technologies and computer tools. We described the knowledge of neural regeneration after heart transplantation, liver transplantation and kidney transplantation and apply them to various imaging techniques to quantify the uptake of radionuclide tracers to assess the prognosis of organ transplantation. We noted that the aim of this review is both to provide clinicians and nuclear medicine researchers with theories and insights into nerve regeneration in organ transplantation and to advance imaging techniques and radiotracers as a major step forward in clinical research. Moreover, we aimed to further promote the clinical and research applications of imaging techniques and provide clinicians and research technology developers with the theory and knowledge of the nerve.

## Introduction

Human organ transplantation is a very significant development in modern medicine. By removing diseased and necrotic organs and replacing them with healthy and viable ones, patients with life-threatening conditions can be given a second chance at life. As of 2019 epidemiological surveys have shown that more than 100,000 patients worldwide need organ transplants each year (Vanholder et al., 2021). Despite the important role of organ transplantation in extending the life of patients, transplanted organs are exposed to multiple risks in the recipient, including modification of the transplantation technique, immune rejection of the transplanted organ, autonomic innervation after denervation of the transplanted organ, and health monitoring of the transplanted organ (Poole et al., 2019). Current organ transplantation techniques include allogeneic, xenogeneic, and future organ transplantations that target highly differentiated cell and tissue transplantation techniques that can minimize immune rejection in the recipient (Sayegh and Carpenter, 2004; Timsit et al., 2019).

Successful organ transplantation is not only dependent on the histocompatibility between the donor and recipient, but also on the functional recovery of the transplanted organ in the recipient tissue, which is one of the criteria for successful transplantation (Patchell, 1994; Byersdorfer and Turnquist, 2021). Nerve regeneration in organ transplantation is the process by which a patient goes from a completely denervated donor organ to a progressive regeneration of autonomic nerves after organ transplantation (Grupper et al., 2018). The most critical aspect of the nerve regeneration process in transplanted organs is the interaction between the Schwann cells and axons. The process of organs transplantations is a process of immune response (Bolívar et al., 2020). Antigens are present in the Schwann cells of the peripheral nerves of the donor organ, which connect to the nerves of the recipient organ and produce a variety of immune factors, resulting in an immune rejection reaction. It is therefore crucial that sufficient immunosuppressive drugs are given during the organ transplantation process to provide a better environment for nerve regeneration in the organ transplant (Sarker et al., 2018).

Autonomic innervation plays a crucial role in maintaining organ function (Li et al., 2021b) and transplanted organs face transient denervation. By performing peripheral nerve anastomoses during transplantation and giving neurotrophic factor drugs postoperatively, the nerves of the transplanted organ have demonstrated that the repair process can be slow. Several studies have shown that denervated organs have a strong regenerative capacity, and this has led to the widespread use of organ transplantation and saving countless lives (White et al., 2015).

**FIGURE 1:**
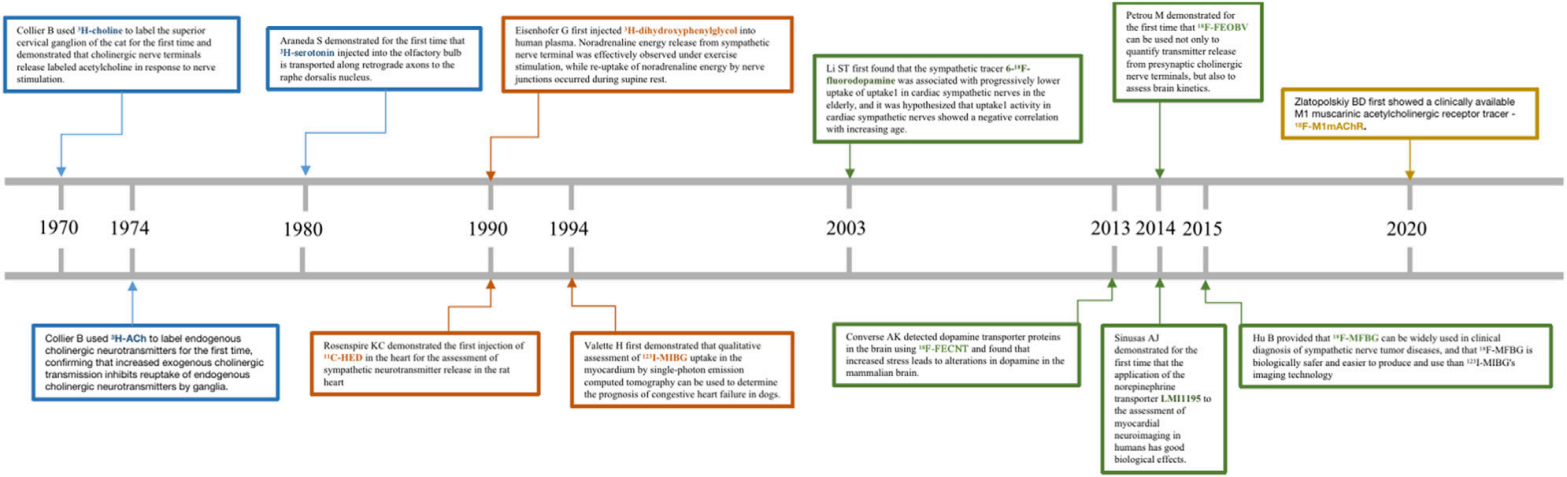
Depicts a timeline of the development of radiotracers.

Scientists were able to further observe the neuroanatomical patterns and their normal physiological functions *in vivo* until the advent of conventional tracers in the 1970s, which completely broke this bottleneck and led to a rapid advancement in neuroscience and cognitive science (Kristensson, 1970). Scientists subsequently exploited the physiological properties of rabies virus transmission on nerve axons to develop viral tracers with specificity and high expression (Ugolini, 2010). In conclusion, both traditional neural tracers and viral tracers (Fan et al., 2020; Feng et al., 2020) have been widely used in scientific investigations to reveal neuronal connections between brain regions, neurotransmission between skeletal muscles or visceral organs and the brain, and to further clarify the neural and functional localization of the cerebral cortex (Huang et al., 2022; Li et al., 2021a). With the development of the field of nuclear medicine, radionuclide testing gradually emerged in the 1990s as a clinical application to measure and monitor blood flow, urine, and neurotransmitter expression in patients (Schwaiger et al., 1990). Among other things, radionuclide tracers can be used to assess the functional status of organs and tissues and to determine the progression and prognosis of a patient’s disease with the greatest precision and speed in the patient. These are the advantages of radiotracers, such as: ① the simplicity and sensitivity of the means of detection; ② the fact that they are in accordance with the normal physiological conditions of the organism and can be metabolized *in vivo*; and ③ the fact that they can be precisely localized and can provide detection at the molecular and atomic level. We have shown in Table 1 the advantages and disadvantages of conventional tracers, viral tracers, and radioactive tracers. In addition, we have depicted in Figure 1 a timeline for the development of radiotracers.

**TABLE 1:** Describes the advantages and disadvantages of traditional neural tracers, viral tracers, and radioactive tracers currently in use.

Species	Product	Advantages	Disadvantages
Traditional neural tracers	Horseradish peroxidase	1 more sensitive	1 Involved in cellular metabolism
	Cholera toxin B		
	Fluoro-Gold	2 Only fluorescent microscopy and electron microscopy are required for observation	2 Unstable and easily degraded.
	Wheat germ agglutinin		3 Restricted conditions of use
Viral tracers	Adeno-associated viral vector	1 High sensitivity, directionality and selectivity compared to traditional neural tracers	1 High cytotoxicity
	Rabies virus	2 Highly infectious	2 For scientific investigation only
	Herpes simplex virus	3 No attenuation of the signal transmission of neural tracers	
Radioactive tracers	Commonly used radioisotopes are carbon-11, nitrogen-13, oxygen-15, iodine-131 and iodine-135	1 High sensitivity, safe and radiation-free	1 The need for a dedicated laboratory
		2 Simple detection means, suitable for *in vivo* experiments and clinical diagnosis	2 The need for specialist technicians
		3 Non-cytotoxic	3 Protection is sometimes required
		4 Conforms to the normal physiological conditions of the organism and is metabolizable	
		5 More precise localization, widely used for cardiovascular diseases and tissues and organs	

**FIGURE 2:**
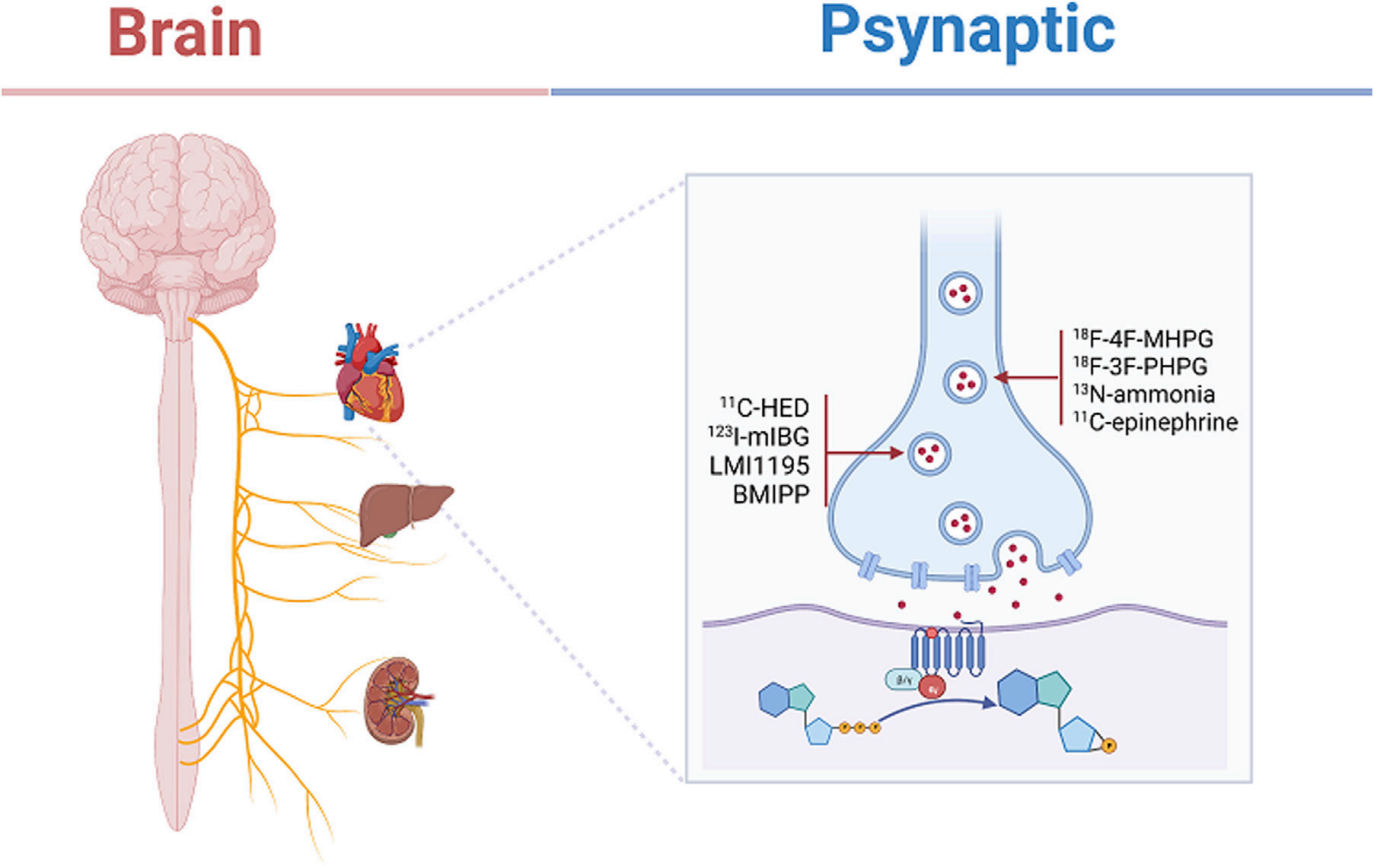
Depicts the present state of sympathetic innervation of transplanted hearts. Labeling of commonly used radioactive neurotransmitters in the presynaptic membrane, such as ^11^C-hydroxyephedrine (^11^C-HED), Iodine-123-meta-iodobenzylguanidine (^123^I-mIBG), *N*-[3-bromo-4-(3-^18^F-fluoro-propoxy)-benzyl]-guanidine (LMI1195), ^123^I-15-(*p*-iodophenyl)-3-*R,S*-menthyl pentadecanoic acid (BMIPP), 4-^18^F-fluoro-meta-hydroxyphenethylguanidine (^18^F-4F-MHPG), 3-^18^F-fluoro-para-hydroxyphenethylguanidine (^18^F-3F-PHPG), ^13^N-ammonia, ^11^C-epinephrine. This figure was created using the biorender application (Biorender.com).

Currently, with the development of imaging technology, the use of neural tracer techniques to visualize the regenerative processes of autonomic nerves and to label neuronal and neurotransmitter neurotransmission can reveal the sympathetic and parasympathetic reinnervation processes in animals and humans (Bravo et al., 2015; Wang et al., 2018). Positron emission tomography imaging (PET) (Brust et al., 2014), single photon emission computed tomography (SPECT) combined with magnetic resonance (MRI) and computed tomography (CT) (Srinivas et al., 2010), involving radiologically targeted molecules to label the chemical neurotransmitters between synapses, such as ^11^C-hydroxyephedrine (^11^C-HED), ^11^C-epinephrine (Sasano et al., 2008), ^11^C-phenylephrine (PHEN) (Raffel et al., 1999), 6-^18^F-fluorodopamine (Goldstein et al., 1993), ^13^N-ammonia, and flubrobenguane (FBBG) (Zelt et al., 2021) has been found to be useful in presynaptic neuronal transport processes in the cardiovascular system. Our review will demonstrate the use of a radiotracer in the reinnervation of the heart, liver, and kidney after transplantation as shown in Table 2. Numerous studies have illustrated the usefulness of radiotracer applications in probing the neuroanatomy of the cardiovascular system, organ transplantation and the nervous system (Bailey and Willowson, 2013). Therefore, we have summarized the current widespread use of nerve tracers in autonomic disorders in Table 3. We showed the innervation of the heart, liver, and kidney in detail in Figure 2, with sympathetic regeneration of the heart transplant as an example, and we have labelled the commonly used sympathetic tracers in the diagram. The image was created using Biorender.com.

**TABLE 2:** The use of clinically available radiotracers in heart and renal transplantation.

Organ transplantation	Nerve tracer	Technology	Nerve reinnervation	Nerve regeneration time	References
Heart transplantation	^11^C-hydroxyephedrine (^11^C-HED)	PET/CT	Sympathetic nerve	1 year	Schwaiblmair et al. (1999); Bengel et al. (2001)
Heart transplantation	^11^C-hydroxyephedrine (^11^C-HED)	PET/CT	Sympathetic nerve	5 years	Uberfuhr et al. (2000)
Heart transplantation	Iodine-123-meta-iodobenzylguanidine (^123^I-mIBG)	SPECT	Sympathetic nerve	2 years	Estorch et al. (1999)
Renal transplantation	Iodine-123-meta-iodobenzylguanidine (^123^I-mIBG)	SPECT	Sympathetic nerve	6 months	Rasmussen et al. (2020)

In clinical studies, Iodine-123-meta-iodobenzylguanidine (^123^I-mIBG) and ^11^C-hydroxyephedrine(^11^C-HED) are the most widely used radionuclide tracers for sympathetic nerve regeneration in cardiac transplants. ^123^I-mIBG, has also shown significant tracer effects in sympathetic nerve regeneration in renal transplants. We found that cardiac sympathetic reinnervation was observed as early as 1 year after heart transplantation and renal sympathetic reinnervation 6 months after renal transplantation.

**TABLE 3:** The targeted molecular tracers currently used clinically in autonomic nervous system diseases.

Application	Nerve tracer	Technology	Target	References
Cardiomyopathy	4-^18^F-fluoro-meta-hydroxyphenethylguanidine (^18^F-4F-MHPG), 3-^18^F-fluoro-para-hydroxyphenethylguanidine (^18^F-3F-PHPG)	PET/CT	Sympathetic nerve	Raffel et al. (2018); Raffel et al. (2022)
Myocardial infarction	^13^N-ammonia,^11^C-epinephrine	PET	Sympathetic nerve	Sasano et al. (2008)
Ischemic cardiomyopathy	Flubrobenguane (FBBG)	PET	Sympathetic nerve	Zelt et al. (2021)
Heart failure	*N*-[3-bromo-4-(3-^18^F-fluoro-propoxy)-benzyl]-guanidine (LMI1195)	PET	Sympathetic nerve	Sinusas et al. (2014); Higuchi et al. (2015); Chen et al. (2018)
Vasospastic angina	Iodine-123-meta-iodobenzylguanidine (^123^I-mIBG),^123^I-15-(*p*-iodophenyl)-3-*R,S*-menthyl pentadecanoic acid (BMIPP)	PET	Sympathetic nerve	Watanabe et al. (2002)
Alzheimer’s disease	(-)-^18^F-fluoroethoxybenzovesamicol (^18^F-FEOBV)	PET	Presynaptic cholinergic	Mulholland et al. (1998); Petrou et al. (2014)
Hepatocellular carcinoma	^14^C-Cho	PET	Choline	Kuang et al. (2010)
Prostate carcinoma, Parkinson’s disease	^11^C-donepezil	PET	Parasympathetic nerve	Gjerløff et al. (2015), Nielsen et al. (2019)

## Reinnervation of a transplanted heart

### Reinnervation of the sympathetic plexus in the transplanted heart

In 2022, 57-year-old David Bennett became the first patient in history to have a pig heart transplant, but passed away 2 months following the allogeneic cardiac transplantation (Burki, 2022). Back in 1947, Dr Christiaan Barnard set the record for the first in orthotopic heart transplant (Brink and Hassoulas, 2009). The first patient, Louis Washansky, passed away 18 days after the operation, but the second patient, Philip Blaiberg, survived for almost 2 years (Cooper, 2001). We are grateful to the pioneer of heart transplantation, the courageous Dr Christiaan Barnard! While orthotopic heart transplantation is now well established, patients who have undergone cardiac transplantation continue to face a variety of post-operative complications and immune rejection reactions, the causes and mechanism of which deserve to be adequately explored.

Heart transplantation is the treatment of choice for patients with end-stage heart failure (Mehra, 2017). In both allogeneic and orthotopic heart transplantation, the donor heart faces denervation and is vulnerable to a variety of cardiovascular events in the absence of central innervation, such as arrhythmias, abnormal chest pain, sudden atrial fibrillation, sudden cardiac death, and stroke (Joglar et al., 2021; Firoz et al., 2022). With the popularity of the heart transplantation approach and extensive research, it has been reported that the sympathetic nerves of the heart gradually regenerate over time, restoring sympathetic innervation to the heart and participating in the rhythm regulation of the heart and the perfusion of the heart muscle (Jakus et al., 2022). The state of cardiac transplant sympathetic reinnervation varies from one heart transplant patient to another, with some heterogeneity in various regions of the heart (Wilson et al., 1991). The phenomenon of reinnervation after heart transplantation is therefore an innovative point of clinical research!

With the technical support of medical imaging, nerve tracers are used to label the major transmitters of the post-sympathetic adrenergic nerves and thus determine the integrity of the sympathetic nerves (Pandit-Taskar and Modak, 2017). ^11^C-HED functions as a catecholamine analogue with a neural tracer effect and is used in combination with a presynaptic norepinephrine transporter (NET, uptake-1) to assess the activity of presynaptic sympathetic neuronal transport in the myocardium (Zelt et al., 2021). When assessing the sympathetic integrity of the heart 1 year after heart transplantation using PET, 55% of patients (16) presented with ^11^C-HED retention in the left anterior descending branch at a rate of 47%. Subsequently, after exercise stress stimulation of cardiac sympathetic fibers, patients with cardiac reinnervation present exhibited an 8% increase in left ventricular ejection fraction (LVEF) compared to denervated patients, with the greatest density of reinnervated sympathetic nerves found particularly in the left anterior interventricular region (Bengel et al., 2001). Thus, reuptake of catecholamine analogues by sympathetic nerve endings in the anterior interstitial region of the heart could provide strong evidence of sympathetic reinnervation of the patient’s heart.

Cardiac autonomic nerves are involved in the neuromodulation of the sinus node and heart rate variability (HRV) is a widely used non-invasive measure of the cardiac sympathetic and parasympathetic activity and can be used as a tool to assess the recovery of the autonomic nervous system after heart transplantation (Ernst, 2017). In one study, which assessed cardiopulmonary reinnervation in patients after heart transplantation between 2.5 and 12 months, it was observed that low-frequency (LF) fluctuations in HRV of heart transplant patients in the supine position up to 5.7 ms^2^ were observed as early as 6 months and was up to 6.0 ms^2^ when LF was tested again 1 year later, showing a significant increase in LF variability with sympathetic nerve regeneration (Christensen et al., 2021a). A positive correlation between exercise capacity and peak oxygen uptake and sympathetic reinnervation was observed in patients, with myocardial ^11^C-HED uptake being twice as high in the reinnervated group compared to the denervated group (Schwaiblmair et al., 1999).

Further labelling of sympathetic nerves using catecholamine analogue tracers revealed a positive correlation between the degree of presynaptic retention of ^11^C-HED in sympathetic nerves and the HRV produced by sinus node innervation. At 5 years’ follow-up of post-heart transplant patients, 58% (22) of patients had a mean ^11^C-HED retention rate of 10.7% in the left ventricle, while LF in patients with sinus node reinnervation in HRV was up to 5.9 ms^2^. This indicates that sympathetic regeneration is a continuous process, with an increase in HRV observed as early as 3.5 months, while 5 years later patients showed a wide distribution of sympathetic reinnervation in the anterior and some lateral regions of the left ventricle (Uberfuhr et al., 2000).

Iodine-123-meta-iodobenzylguanidine (^123^I-mIBG), a sympathetic blocker guanethidine (Zhang et al., 2014), is a pseudo neurotransmitter analogue released from the presynaptic membrane of sympathetic nerves, bound to uptake-1 at presynaptic terminals labeled and stored in vesicles, and used to assess presynaptic sympathetic neuronal vesicle storage activity (Mabuchi et al., 2005). In a study, the heart to mediastinal rate ratio (H/M) and the elution rate of the heart were determined using SPECT assessment 4 h after injecting ^123^I-mIBG into the body (Dilsizian and Chandrashekhar, 2022). In patients with allogeneic heart transplants, increased ^123^I-mIBG uptake by cardiac sympathetic nerves occurred in 80% of patients 10 years after heart transplantation compared to patients 2 years after heart transplantation. Of these, sympathetic reinnervation occurred as early as 2 years after allogeneic heart transplantation, at which time cardiac sympathetic reinnervation of nerve fibers to ^123^I-mIBG uptake can be as high as 18% (Estorch et al., 1999). Another study followed up patients after ectopic heart transplantation and found that ^123^I-mIBG scintigraphy remained absent 6 years after ectopic heart transplantation under the detection of SPECT (Yap et al., 2006). It is hypothesized that non-anatomical heart transplantation may result in limited regeneration of cardiac sympathetic fibers. However, Jenkins et al. found that ^123^I-mIBG scintigraphy assessed sympathetic regeneration in patients and that sympathetic reinnervation did not correlate with circadian blood pressure regulation in patients. This suggests that ^123^I-mIBG scintigraphy only confirms the presence of nerve regeneration after cardiac transplantation and that cardiac sympathetic function had not been fully restored (Jenkins et al., 1997).

A recent study reported that quantitative tracers of cardiac sympathetic nerves, 4-^18^F-fluoro-meta-hydroxyphenethylguanidine (^18^F-4F-MHPG) and 3-^18^F-fluoro-para-hydroxyphenethylguanidine (^18^F-3F-PHPG), can be used to quantify nerve density in denervated regions of patients with ischemic myocardial infraction. It is thus, reasonable to speculate their future application in the exploration of full neurological recovery in patients after heart transplantation (Raffel et al., 2022).

### Reinnervation of the parasympathetic plexus in the transplanted heart

In 1995 researchers focused their attention on parasympathetic imaging techniques, using the stimulating effect of meglumine diatrizoate contrast agents on cardiopulmonary chemoreceptors. The heart rate was seen to decrease in heart transplant patients after chemical tracer infusion, which could be inferred from the intact and regenerative parasympathetic innervation of the patients. This study revealed that 6 years after heart transplantation, patients did not only have the parasympathetic reinnervation that the investigators expected, but the rate of residual recipient sinus nodes (RSN) in heart transplant patients showed an increasing trend. This probably occurred without remodeling of the parasympathetic efferent nerves after surgery (Arrowood et al., 1995). The researchers then injected norepinephrine into heart transplant patients to stimulate their pressure receptors to observe changes in cardiac vagal tone, and 4 years after transplantation the patients still did not show increased vagal tone (Arrowood et al., 1997).

Although the results of earlier clinical studies have been unsatisfactory, the latest findings show that cardiopulmonary receptors are reinnervated 1 year after heart transplantation. When Wyller et al. evaluated the autonomic activity of the heart, they found that patients showed an increase in LF in the supine position to 5.7 ms^2^ as early as 6 months after transplantation and a trend towards a decrease in heart rate (HR) at rest 12 months after transplantation. During the 20° head-up tilt test the patient showed a decrease in right atrial pressure and a decrease in cardiac output as HR increased. These findings support the idea that cardiopulmonary receptors are subject to cardiac parasympathetic reinnervation 1 year after transplantation (Wyller et al., 2021).

In physiological studies, autonomic transection of the heart transplant leads to vagal denervation of the heart, manifested by the resting tachycardia and variable hourly cardiac malfunction in patients after heart transplantation (Levy et al., 1981). In one study that followed up cardiopulmonary exercise tests in heart transplant patients, cardiac sinus node parasympathetic regeneration could be observed in year two. There was an increase of up to 6.0 ms^2^ in high-frequency (HF) fluctuations in the HRV index, and an LF/HF radio of up to 84% plus an increase in tachycardia response during Valsalva exercise in patients in the supine position. It is thus, evident that parasympathetic reinnervation begins to gradually strengthen 2 years after heart transplantation (Christensen et al., 2021b).

With the development of neural tracers, chemical tracers acting on cardiopulmonary receptors are no longer sought singularly but are combined with PET techniques to observe the release of acetylcholine (ACh) transmitters following parasympathetic activation (Khajehali et al., 2018). However, unlike neural tracers of catecholamine analogues, Ach is subject to specific degradation by acetylcholinesterase (AChE), which results in more challenging imaging techniques for observing cardiac parasympathetic nerves. As a result, non-invasive HRV testing is predominantly used clinically for cardiac parasympathetic reinnervation. (-)-^18^F-fluoroethoxybenzovesamicol (^18^F-FEOBV) binds to acetylcholine transporter proteins and is prominently labelled at cholinergic nerve endings in the heart. Although ^18^F-FEOBV has not been explored to date for postoperative parasympathetic regeneration in cardiac transplant patients, it is expected to be a tool for future use in cholinergic radiological studies (Petrou et al., 2014).

### Reinnervation of the sensory plexus in the transplanted heart

As a result of heart transplantation, the heart is denervated by sensory fibers. Once a heart transplant recipient develops ischemic angina, the patient’s presentation is highly atypical, making the diagnosis more difficult for the clinician (DeFilippis et al., 2017). Statistics suggest that during the 5-year period after heart transplantation, post-transplant cardiac allograft vasculopathy (CAV) was present in 50% and coronary arteriosclerosis in 10% of patients (García-Baizán et al., 2021).

Research has also shown that heart transplant patients present with pain in the anterior thoracic region after 3 years and stenosis occlusion of the coronary arteries is seen on the coronary computed tomography angiography (CTA). The patient with heterogeneity of coronary stenosis, which occurs mainly in the left anterior descending coronary artery results in complete obstruction of the right coronary vessel. At the same time, post-transplant in the anterior thoracic region has been associated with sensory nerve regeneration in some cases and not in others, and this regeneration often has a negative effect, hence, modulating and reconstructing nerve regeneration in the transplanted heart has great potential for clinical research (Stark et al., 1991)!

## Reinnervation of a transplanted liver

### Reinnervation of the sympathetic plexus in the transplanted liver

Liver transplantation is one of the treatments for chronic liver failure and hepatocellular carcinoma (HCC) (Tan et al., 2022). The liver has a unique ability to regenerate and the donor liver can return to normal liver morphology after 2 months, but research is still needed on the pattern and mechanism of regeneration and innervation of the liver (Haga et al., 2008). The loss of autonomic innervation of the transplanted liver and the progressive decrease in catecholamine release from the hepatic denervation leads to an increase in hepatic blood flow (HBF) through a reduction in action alpha-adrenergic receptors. However, the change in HBF is not significant and therefore the restoration of HBF cannot be used as a decisive indicator of hepatic nerve regeneration (Kurosawa et al., 2002).

As the liver is denervated, the autonomic nervous system gets out of balance with respect to hepatic glucose uptake, and the net hepatic glucose uptake (NHGU) is no longer inhibited by sympathetic nerves. This process can result in an imbalance between the liver’s food uptake function and the body’s postprandial glucose regulation, hence the manifestation of the metabolic syndrome in liver transplant patients (Myers et al., 1991; Moore et al., 2012).

Clinical studies have reported that during the 3–5 years following liver transplantation, 50% of patients are susceptible to metabolic syndrome (MetS), obesity and type 2 diabetes as a result of immunosuppression and liver denervation (Becchetti et al., 2020). Thus, monitoring the computed tomography attenuation values (CT-AV) of liver transplant patients 6 months after surgery revealed that patients with values of CT-AV below 60% in the 1-week postoperative group showed signs of impaired liver function at 6 months after surgery due to steatosis or ballooning of the transplanted liver. It was therefore, hypothesized that CT-AV quantification at 1 week following liver transplantation could be used to assess the prognosis and regeneration of liver transplants (Iida et al., 2005). Liver transplant reinnervation is thus, an issue of scientific interest, both from the perspective of exploring the mechanisms of liver transplant nerve regeneration and improving the complications following liver transplant denervation.

Kjae et al. studied sympathetic nerve regeneration after liver transplantation and established two liver groups: a liver transplantation group (*n* = 13, <30 months after transplantation) and a normal control group (*n* = 11, normal individuals without liver transplantation). When both groups were subjected to liver biopsy for catecholamine levels, the norepinephrine concentration in the liver transplantation group was found to be only 0.022 nmol/g, and the levels of catecholamines 99% lower compared to normal controls, showing that sympathetic regeneration of liver sympathetic nerves was still not present 2–3 years after live denervation (Kjaer et al., 1994).

In rodent studies, direct observation of liver sections from 3 to 6 months after liver transplantation in rats using immunohistochemistry revealed a positive trend for growth-associated protein 43 (GAP-43) as a marker of neuronal plasticity over time. However, nerve regeneration in the liver portal vein occurred only between 5 days and 3 months after liver transplantation and had ceased in the liver by 6 months. Meanwhile, the ubiquitin hydrolase protein gene product 9.5 (PGP9.5) expression in nerve axons had returned to normal levels (Kandilis et al., 2014).

The results observed from rodents demonstrate that nerve regeneration in the liver is completed at 3–6 months, and in the available human studies reported, some investigators used the same immunohistochemical technique to determine the regeneration of liver nerves in humans by direct observation of nerve regeneration (Boon et al., 1992). Fifteen months after liver transplantation, immunostaining for PGP9.5 was found to be positive on liver sections from transplanted patients, but the expression of nerve regeneration appeared restricted, with positive expression only at portal nerve fibers (Dhillon et al., 1992).

### Reinnervation of the parasympathetic plexus in the transplanted liver

Several studies have confirmed that regeneration of Schwann cells wraps around axons after peripheral nerve injury (PNI) and that vagus nerve regeneration is similarly repaired and remyelinated by Schwann cells distal to axons (Clements et al., 2017). Animal studies have revealed that Netrin-1, a laminar adhesion-associated protein expressed in Schwann cells and axons of motor and sensory neurons, targets and activates the Netrin-1 signaling pathway in Schwann cells to promote the regeneration function of peripheral nerve cells after PNI(Taïb et al., 2022). Wang et al. demonstrated that exogenous supplementation of Netrin-1 in mice after liver transplantation accelerated regeneration of the hepatic vagus nerve. Netrin-1 expression was significantly reduced in the liver tissue of mice after liver resection (*p <* 0.05). Subsequently, exogenous supplementation of Netrin-1 was administered in the tail vein of mice and positive expression of GAP-43 was observed in the liver tissue 1 week after surgery, as evidenced by the nerve regeneration in the liver 1 week after Netrin-1 administration. Subsequent detection of positive anti-choline acetyltransferase (ChAT) in the liver tissue further confirmed that Netrin-1 promotes vagal nerve regeneration in the liver (Wang et al., 2022). It is certainly a revelation to us that the research and development of exogenous targeted neurological drugs for post-liver transplant patients point to pharmaceutical innovation that will help in the rehabilitation of liver transplant patients with neurological regeneration.

It is well known that the central nervous system plays an important physiological regulatory role on the autonomic nerves of the liver (Liu et al., 2021). Neuro-humoral regulation of the liver is mediated through sympathetic afferents to the central nervous system, with projections in the lateral hypothalamus (LH) and ventral medial hypothalamus (VMH) to the dorsal motor nucleus of the vagus (DMV) and subsequent projections to primary neurons involved in hepatic vagal innervation (Berthoud and Neuhuber, 2019). An experiment involving rats showed that partial hepatectomy with concomitant destruction of VMH in a rat’s brain resulted in an increase in DNA synthesis in the liver. This probably led to compensatory stimulation of hepatocyte regeneration by the hepatic vagus nerve as a result of disruption of the central nervous system (Kiba et al., 1994). The hepatic vagal branch has an independent innervation function on DNA synthesis in hepatocytes, and once liver denervation has occurred, this results in a delayed effect on DNA synthesis in hepatocytes (Kato and Shimazu, 1983; Kiba et al., 1995). David et al. observed that the hepatic vagus nerve secretes ACh, which acts on muscarinic acetylcholine receptor 3 (mAChR3) in hepatic progenitor cells (HPCs) to promote hepatocyte regeneration. Rats with hepatic vagal branches removed showed impaired proliferation of hepatocytes and bile duct epithelial cells (Cassiman et al., 2002). It is clear that hepatic parasympathetic denervation leads to a decrease in the rate of DNA synthesis in hepatocytes and thus interferes with hepatic regeneration. The rate of DNA synthesis in hepatocytes is reduced by parasympathetic denervation of the liver, thereby interfering with liver regeneration.

In the cytoplasm of nerve cells, presynaptic ChAT acts to synthesize ACh from choline (Cho) and acetyl coenzyme A (CoA), which binds to muscarinic receptors and participates in cholinergic signalling (Sarter and Parikh, 2005). The use of the radiotracer ^14^C-Cho to label the synthesis of hepatocyte membranes and the process of acetylcholine transmitter synthesis has been found, and it is hypothesized that ^14^C-Cho could be used as a diagnostic for new-onset HCC and recurrence after liver transplantation (Kuang et al., 2010). In contrast to neural regeneration in heart transplantation, patients who have undergone liver transplantation for HCC are at a risk of HCC recurrence over a 5-year period (Agopian et al., 2015). Despite the temporary lack of studies on hepatic vagal reinnervation, it is suggested that studies using nerve tracers to monitor cancer recurrence in patients after transplantation are more clinically relevant and have a highly complementary diagnostic role for prognosis after liver transplantation (Kim et al., 2020).

## Reinnervation of a transplanted kidney

### Reinnervation of the efferent nerves in the transplanted kidney

According to literature, as of November 2021, surgeons have transplanted alpha-gal knockout porcine kidneys into brain-dead patients. The results indicate that after 54 h of *in vivo* renal filtration function of the allogeneic kidneys, the patients had yet to experience significant immune rejection, showing the great strides being made today with allogeneic kidney retransplantation (Cooper and Hara, 2021; Dolgin, 2021).

Kidney transplantation is the most effective treatment for end-stage renal disease (ESRD) and is effective in improving the survival of patients with renal failure, with an estimated survival of 19.2 years for kidney transplant patients (Poggio et al., 2021). In anatomical studies, the kidney is innervated by the sympathetic ventral plexus emanating from T12-L2 spine cord, and the nerves of the kidney are involved in innervating water and sodium metabolism and fluid regulation in the kidney (Sakakura et al., 2014). Interruption of renal sensory afferent and sympathetic efferent nerves after renal transplantation leads to inactivation of sympathetic efferent nerve effectors, which in turn activate the renal-renal reflex and inhibit the renin-angiotensin-aldosterone system. This process implies the development of reduced renin secretion, diuresis and pro-sodium excretion, and the lowering of blood pressure (Frame et al., 2016).

In animal experiments, renal denervation was shown to reduce blood pressure in rats with nephrogenic hypertension. Compared with rats with hypertension caused by renal artery ligation, renal denervation restored normal plasma renin secretion, reduced arterial blood pressure by 44 ± 3 mmHg and reduced HR by 33 ± 9 to 61 ± 9 beats per minute, showing that renal denervation improved the symptoms of nephrogenic hypertension (Oliveira et al., 1992). One study used myocardial uptake of ^123^I-mIBG to measure autonomic recovery after renal transplantation. They reported a reduced elution rate of ^123^I-mIBG for cardiac sympathetic nerves assessed 3 months after renal transplantation (*p* <0.05 ) and no differential change in the assessment of HRV in patients. It is proposed that sympathetic overexpression due to ESRD was reduced after renal transplantation and that the ^123^I-mIBG uptake rate could be used as a specific indicator of renal denervation (Kurata et al., 2004).

Given the involvement of renal sympathetic over-activation in the pathophysiology of hypertension, the implementation of renal sympathetic denervation (RDN) is suggested to be an effective treatment for intractable hypertension (Mahfoud et al., 2022; Sarathy and Salman, 2022). In the Global SYMPLICITY Registry project, the Symplicity flex catheter was used to examine ambulatory blood pressure changes in 3,000 patients 3 years after RDN. The results showed a decrease in systolic blood pressure (SBP) starting 6 months after RDN (−11.7 ± 28.6 mmHg, *p <* 0.001) which continued to decrease (−16.6 ± 28.6 mmHg, *p <* 0.001) (Mahfoud et al., 2019). However, experiments in animals have revealed a regeneration of renal nerves after RDN. Findings at 5.5 months after RDN in sheep, showed positive tyrosine hydroxylase (TH) staining in the perirenal tissue, while renal norepinephrine levels were detected reaching 88.9% and 131.0% after 11 months of RDN. These results suggest that nerve regeneration in the perirenal artery 5.5 months after RDN and innervation could return to normal levels after 11 months. But this study is limited to sheep with ductal RDN and may serve as a partial reference for renal denervation in humans (Booth et al., 2015).

In a pathological study, Gazdar et al. used immunohistochemistry to detect renal nerve fibers by observing patients from 5 to 3,012 days after renal transplantation. Positive Bodian staining of regeneration axons were observed as early as 28 days after renal transplantation, and substantial nerve regeneration around the axons after 8 months (Gazdar and Dammin, 1970). Similarly, Rabelink et al. studied renal sympathetic regeneration in allogeneic transplanted kidneys 2 months later, and compared a healthy control group with a renal transplant group for head-out water immersion (HOWI). They found a marked increase in urinary sodium and potassium concentrations in renal transplant patients. It is speculated that 2 months after renal transplantation the kidney is still denervated and that the reduction in renal vascular resistance led to transplant patients exhibiting diuresis and natriuresis (Rabelink et al., 1993).

Mauriello et al. followed up patients for 5 months to 11 years after renal transplantation and found that nerve regeneration could be observed in patients at 5 months of transplantation, with positive staining for the neuronal regeneration and GAP-43 seen in the renal arteries. In contrast, 10 years after transplantation in patients with concomitant hypertension, TH was detected in renal tissue positively labelled in sympathetic nerve fibers. It is hypothesized that the patients experienced regeneration of renal sympathetic nerves in response to hypertension stimulation (Mauriello et al., 2017). Nevertheless, hypertension after renal transplantation is not associated with regenerative innervation of the renal arteries. Grisk et al. reported a remarkable increase in mean arteria pressure 3 weeks after renal transplantation, but TH mRNA levels in sympathetic nerve fibers were not yet significantly altered. This demonstrates that sympathetic nerves were not yet regenerated 3 weeks after renal transplantation and that post-transplant hypertension was not neurogenic (Grisk et al., 2000). In addition, Rasmussen et al. observed a 30% reduction in ^123^I-mIBG uptake by the transplanted kidney 1 month later compared to ^123^I-mIBG uptake by the donor kidney within 4 h preoperatively (*p <* 0.005). This illustrates a positive correlation between recovery of renal function in the transplanted kidney and renal reinnervation (Rasmussen et al., 2020).

### Reinnervation of the afferent nerves in the transplanted kidney

It has been reported that renal sensory afferent nerve cells originate from the dorsal root ganglion (DRG) of the ipsilateral T10-L2, mostly located in the cortical spinal cord area and the renal pelvic wall (Marfurt and Echtenkamp, 1991; Ma et al., 2002). Increased ureteral pressure stimulates pressure receptors and chemoreceptors in the kidney and increases the release of calcitonin gene-related peptide (CGRP) and substance P (SP) in the renal pelvis tissue. This activates the afferent nerve in the ipsilateral kidney and the renal-renal reflex in the contralateral kidney for contralateral urination and natriuretic response (Kopp and Smith, 1989). As the sensory afferent nerves of the kidney are involved in the neuromodulation of renal chemoreceptors, mechanoreceptors and nociceptive receptors, once renal denervation has occurred, renal denervation of afferent nerves leads to impaired renorenal reflexes in the kidney (DiBona and Kopp, 1997; Barry and Johns, 2015). Based on the importance of renal afferent nerves in physiological studies, it is worth exploring the reinnervation of renal afferent nerves.

Rodionova et al. investigated the regeneration process of unilateral renal denervation by measuring the levels of the neural markers TH and CGRP in rat kidney tissue sections. Compared to 1 week after denervation, rats exhibited a marked increase in CGRP and TH levels 3 months after surgery, which showed some degree of regeneration of afferent and efferent nerves in the kidney after 3 months. Meanwhile, the fluorescent tracer 1,1′-dioctadecyl-3,3,3′,3′tetrameylindocarbocyanine methanesulfonate (Dil) was injected into the ipsilateral DRG cells of the kidney. The results confirmed that ipsilateral renal denervated afferent nerve regeneration is not positively correlated with the innervation of the contralateral afferent nerve (Ai et al., 2007; Rodionova et al., 2016). The same study, which confirmed denervation in sheep, found that CGRP levels in renal tissues returned to normal 11 months after RDN (Booth et al., 2015). Interestingly, there is species heterogeneity in the reinnervation of renal afferent nerves (Mulder et al., 2013; Booth et al., 2015). While anatomical and biochemical studies currently provide support for renal afferent sensory reinnervation, no definitive human studies have been reported and further clinical trials are needed to address these questions.

## Conclusion

Transplanted organs face reinnervation of autonomic nerves. This review on neural regeneration in organ transplantation analyses the use of molecularly targeted imaging techniques and immunohistochemical methods that probe the autonomic nervous system of parenchymal organs such as the heart, liver, and kidney, further supporting the reinnervation of those autonomic nerves in transplanted organs.

In our review of the latest radiotracers for neural regeneration in organ transplantation, we found that the development of radiotracers for sympathetic catecholamines and catecholamine analogues is currently dominated by the development of acetylcholinergic tracers. In addition, the development and application of acetylcholinergic tracers cause a chemical reaction that make the molecules susceptible to decomposition and instability. This could be the reason why the current exploration of neural tracers for parasympathetic nerves is still in the early stages. Despite this negative impact, the use of radiotracers in neuroscience has greatly improved our neurological understanding of the central and peripheral nervous systems. In an environment where diagnostic molecular tracers are showing a spurt in development as the means of imaging technology continue to evolve, it is on the basis of the unique advantages of radioactive tracers themselves in accurately identifying nerve reinnervation that we need to be more refined and quantified in organ transplantation research. Here we find out that the *N*-[3-bromo-4-(3-^18^F-fluoro-propoxy)-benzyl]-guanidine (LMI1195) tracer is expected to be one of the most promising sympathetic tracers, and existing studies have successfully assessed the heterogeneity of cardiac innervation in patients by quantifying the myocardial uptake of norepinephrine transporter protein in specific regions (Sinusas et al., 2014; Higuchi et al., 2015). Another novel tracer, ^11^C-GMO, has an excellent kinetic uptake rate and has been shown to have a half-life of 217 h in myocardial sympathetic neurons of rats, 100 times the half-life of ^123^I-mIBG. It is the stable and prolonged metabolism of ^11^C-GMO that can be used to finely measure early cardiac sympathetic reinnervation processes (Raffel et al., 2013). We therefore venture to hypothesize that future studies could focus on quantifying the density of regenerating nerves in transplanted organs, which could help predict the prognosis and diagnostic assessment of organ transplant patients. In conclusion, our review elucidates the reinnervation of heart, liver, and kidney transplants with the support of imaging techniques, providing clinicians with diagnostic tools and guidelines in the field of nerve regeneration.
